# Stereotaxic cutting of *post-mortem* human brains for neuroanatomical studies

**DOI:** 10.3389/fnana.2023.1176351

**Published:** 2023-05-18

**Authors:** Miguel Ángel García-Cabezas, Isabel Pérez-Santos, Carmen Cavada

**Affiliations:** ^1^Department of Anatomy, Histology and Neuroscience, School of Medicine, Universidad Autónoma de Madrid, Madrid, Spain; ^2^Ph.D. Program in Neuroscience Cajal-UAM, School of Medicine, Universidad Autónoma de Madrid, Madrid, Spain; ^3^Neural Systems Laboratory, Department of Health Sciences, Boston University, Boston, MA, United States

**Keywords:** stereotaxis, stereotaxic instrument, Talairach, human neuroanatomy, intercommissural plane

## Abstract

Stereotaxis is widely used in clinical neurosurgery, neuroradiosurgery, and neuroimaging. Yet, maps of brain structures obtained from *post-mortem* human brains are not usually presented in known stereotaxic coordinates. *Post-mortem* brain data given in stereotaxic coordinates would facilitate comparisons with *in vivo* human neuroimages and would also facilitate intra and inter-experiment comparisons. In this article, we present a crafted instrument for stereotaxic cutting of *post-mortem* human brain hemispheres. The instrument consists of a transparent methacrylate plate facing a mirror, four legs, and lateral regularly spaced columns permitting the insertion of large knives in-between the columns. This instrument can be built in any laboratory to obtain human brain slabs in the stereotaxic space of Talairach and Tournoux. We explain in detail the procedure for stereotaxic cutting of human brain hemispheres in the coronal plane, as well as the basis for calculating stereotaxic coordinates of histological sections obtained following the stereotaxic cutting protocol.

## 1. Introduction

Stereotaxic (or stereotactic) is a term that refers to procedures “*characterized by precise positioning in space; said especially of discrete areas of the brain that control specific functions*”; it is usually applied to “*types of brain surgery that use a system of three-dimensional coordinates to locate the site to be operated on*” (Dorland, 2007). Thus, stereotaxis aims to precisely localize a brain point using three coordinates that are defined as the distance of this point to the three space reference planes (X, Y, and Z). Currently, stereotaxis is regularly used in clinical neurosurgery [including deep brain stimulation, deep electrophysiological recording, and tumor biopsy (Pouratian and Sameer, 2020)], clinical neuroradiosurgery (Sheehan and Lunsford, 2022), and experimental brain research in laboratory animals [e.g., injection of axonal tracers (Joyce et al., 2020; Timbie et al., 2020)].

The history of stereotaxis and its applications to brain research and human neurosurgery has been summarized in several extensive reviews (Gildenberg and Krauss, 2009; Rahman et al., 2009; Rzesnitzek et al., 2019); thus, we briefly mention its most relevant hallmarks. The first stereotaxic apparatus for brain surgery was designed by Horsley and Clarke (1908) with the purpose of producing brain lesions in laboratory animals to study synaptic connections by means of secondary degeneration (Sachs, 1909). In the Horsley and Clarke apparatus, the desired targets in the animal brain were located according to external (skull) fiduciary marks. This led to frequent target errors because the anatomical correlation of brain structures with skull landmarks is poor. Therefore, neurosurgeons could not implement stereotaxic approaches until brain targets could be aimed in relation to landmarks inside the brain itself (Rahman et al., 2009). This was achieved by Spiegel et al. (1947) who used a system of coordinates based on the radiological identification of the ventricles (using pneumoencephalograms) and the pineal gland (identified by its characteristic calcifications in regular roentgenography).

A consequence of the development of human stereotaxic neurosurgery was the elaboration of *post-mortem* human brain stereotaxic atlases. These atlases were meant to help neurosurgeons to precisely locate brain targets in relation to the described intracerebral references [the features and evolution of human brain stereotaxic atlases have been described in detail by Coffey (2009)]. The most commonly used stereotaxic space in human brain atlases (*e.g.*, Schaltenbrand and Bailey, 1959) relies on a line joining the anterior commissure (AC) and the posterior commissure (PC). This is the criterion followed by Talairach et al. (1957, 1993), Talairach (1967), and Talairach and Tournoux (1988), who set specific guidelines for accurate alignment of brain hemispheres to the AC and PC. Talairach realized that the deep brain structures of interest for stereotaxic neurosurgery [*e.g.*, nucleus VIM -ventral intermediate- in the thalamus (Spiegel et al., 1952)] have a consistent anatomical relation with an intercommissural line passing through the superior edge of the AC and the inferior edge of the PC. Thus, the Talairach space defines three planes: the midsagittal plane, the horizontal commissural plane perpendicular to the midsagittal plane, and a coronal plane passing through the posterior edge of the AC and perpendicular to the other two planes (Talairach and Tournoux, 1988). These precise guidelines for the alignment of the brain using the AC and PC anatomical references followed by Talairach and Tournoux are more specific and reproducible than those of other human atlases that also use AC and PC as main references, like that of Schaltenbrand and Bailey (1959). The latest editions of the Talairach atlas (Talairach and Tournoux, 1988; Talairach et al., 1993) incorporated brain imaging, making its stereotaxic coordinates the most widely used in human brain mapping (Mazoyer, 2008; Cox, 2012; Makris et al., 2017).

Stereotaxic approaches can also be applied to human neuroanatomy by cutting *post-mortem* human brains within a predetermined stereotaxic space. This approach allows for obtaining thin brain sections that, once processed for molecular markers of interest, can be mapped in known stereotaxic coordinates to facilitate comparison with *in vivo* brain imaging (e.g., García-Cabezas et al., 2007). In spite of this advantage, the most relevant human neuroanatomical studies of the last decades have been performed in manually cut *post-mortem* human brains without stereotaxic reference (e.g., Hirai and Jones, 1989). In the present article we describe a stereotaxic apparatus that was developed in our laboratory for cutting *post-mortem* human brains in the Talairach and Tournoux stereotaxic space. We provide indications to build a similar apparatus in other institutions and exemplify its use to obtain stereotaxic coronal slabs of *post-mortem* human brains.

## 2. Materials and equipment

As far as we know, there is no commercial human stereotaxic frame for cutting *post-mortem* brains in stereotaxic planes. Thus, we designed in our laboratory a crafted instrument for stereotaxically cutting human brains in the space of Talairach and Tournoux.

**FIGURE 1:**
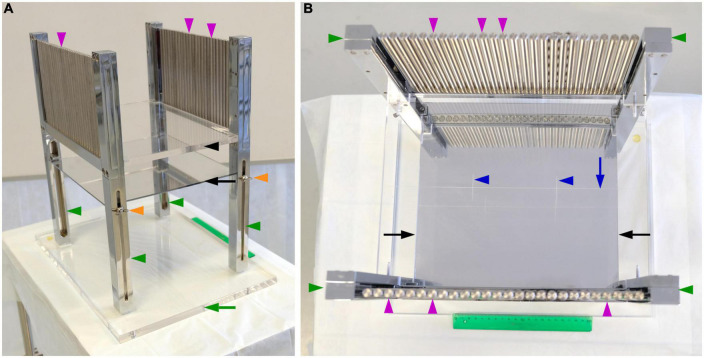
Instrument for stereotaxic cutting *post-mortem* human brains. **(A)** Photograph of a side view of the instrument showing its components. **(B)** Photograph of an overhead view of the instrument, showing its components and the carved lines to align the brain. Components of the instrument as labeled in the figure: Methacrylate plate, black arrowhead; mirror, black arrow; methacrylate base, green arrow; metal legs, green arrowheads; metal columns perpendicular to the methacrylate plate, magenta arrowheads; screws to adjust the vertical position of the mirror, orange arrowheads; long line carved onto the methacrylate, blue arrow; short carved lines onto the methacrylate plate, blue arrowheads.

**FIGURE 2:**
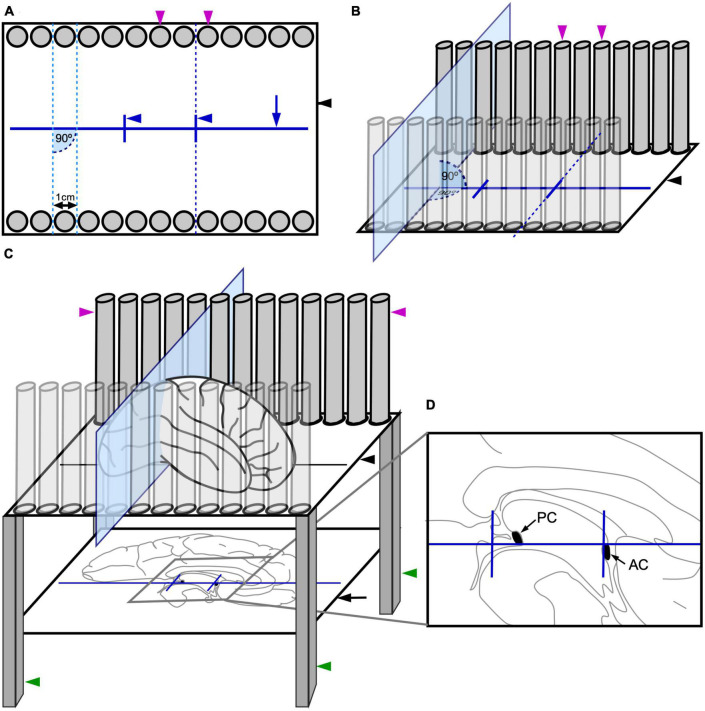
Sketches of the instrument for stereotaxic cutting *post-mortem* human brains. **(A)** Overhead view sketch of the methacrylate plate (black arrowhead) and the metal columns (magenta arrowheads), showing the spatial relations between the metal columns, the carved lines (navy blue solid lines), and the planes created by facing slots between the metal columns (sky-blue and navy blue dashed lines). Note that, for simplicity, only 13 metal columns have been represented on each side in these sketches, instead of the 26 of the real instrument (Figure 1). **(B)** Side sketch of the methacrylate plate and the metal columns, showing the spatial relations between the elements and one of the planes created by facing slots between the metal columns (sky-blue plane). **(C)** Side sketch of the complete instrument, including the methacrylate plate (black arrowhead), the metal columns (magenta arrowheads), the mirror (black arrow), and the legs (green arrowheads); with a brain placed on the instrument. **(D)** Detail of the medial face of the brain hemisphere reflected on the mirror, with the anterior (AC) and posterior (PC) commissures intensified and aligned with the lines carved on the methacrylate plate (drawn in navy blue).

The instrument consists of a transparent methacrylate plate (black arrowhead, Figure 1A) of 30 cm× 25 cm× 1 cm, a mirror (black arrow, Figures 1A, B) of 25 cm× 24.5 cm, four metal legs (green arrowheads, Figures 1A, B) of 2.5 cm× 2.5 cm× 40 cm, 52 metal columns (magenta arrowheads, Figure 1B) of 14 cm height and 0.8 cm in diameter, and a methacrylate base (green arrow, Figure 1A) of 40 cm× 30 cm× 1.5 cm. We describe the spatial relations of all these elements using the cartoons of Figure 2.

First, there is a horizontal transparent rectangular methacrylate plate to hold the brain hemisphere (black arrowhead, Figures 2A–C) supported by four metal legs (green arrowheads, Figure 2C), like a small table. This transparent plate permits visualization from below of structures leaning onto it. The methacrylate plate presents three carved lines that help orienting the brain hemisphere. A long line is carved along the central portion of the methacrylate plate in parallel to its long axis (navy blue arrow, Figures 1B, 2A). Two additional short lines are carved on the methacrylate plate crossing perpendicularly the long line at approximately 1/3 and 2/3 of its length (navy blue arrowheads, Figures 1B, 2A).

Second, the methacrylate plate holds on a series of 26 vertical metal columns (magenta arrowheads, Figures 2A–C) on each of its long margins. The columns (0.8 cm in diameter) form 90° angles with the methacrylate plate, are aligned in parallel to the long carved line, and are separated by open slots of 0.2 cm. Thus, the distance between the center of two consecutive slots is 1 cm (0.8 cm column diameter + 0.2 cm slot). Each column faces another column on the opposite margin of the methacrylate plate. Imaginary planes pass through the slots between two “face-to-face” pairs of columns on each plate margin (sky-blue dotted lines, Figure 2A; sky-blue plane, Figures 2B, C). These imaginary planes are perpendicular to the long carved line (90° angles in Figures 2A, B), and run parallel to the short carved lines. Thus, the distance between two consecutive imaginary planes is 1 cm. Two of those planes pass along the short carved lines (navy blue dotted line, Figures 2A, B).

Third, the legs of the instrument also support a mirror that is placed beneath the methacrylate plate facing upward (black arrow, Figure 2C). This mirror permits that the structures on the methacrylate plate are reflected and seen by the experimenters while placing and cutting the brain. Thus, the long and short lines carved on the methacrylate plate as well as the medial surface of a brain hemisphere resting on the plate are reflected on the mirror (Figure 2C). This allows for precise alignment of hallmarks in the medial face of the hemisphere (AC and PC) with the carved lines (Figure 2D). The heigh of the mirror can be modified by four adjustable screws, one in each leg (orange arrowheads, Figure 1A).

The slots between the metal columns permit the insertion of a sharp blade through them, as shown by the sky-blue plane in Figures 2B, C. The blades are custom-made, 1.5 mm thick; their cutting edge is of the Scandinavian or V grind type (symmetrical, V shaped, sharped cutting edge). Therefore, the cutting edge of the blade cuts through the central plane of the slot and the distance between two consecutive cutting planes is 1 cm. The orientation of the blade passing through the slots is perpendicular to the methacrylate plate and to the long carved line (90° angles in Figures 2A, B). While stored, the blades are kept cushioned onto polyurethane foam to minimize blunting of the cutting edge. After each use, the knifes are cleaned with running tap water, rinsed in ethanol, and dried with absorbent paper. When necessary, blades are sharpened by a professional knife grinder.

## 3. Methods

We exemplify the use of the instrument for human brain stereotaxic cutting using one *post-mortem* human brain obtained from the Program of Body Donation for Teaching and Research of the School of Medicine of Autónoma University of Madrid; the use of this brain for the present study was approved by the Ethics Committee for Research of Autónoma University of Madrid (Authorization CEI-104-2011).

**FIGURE 3:**
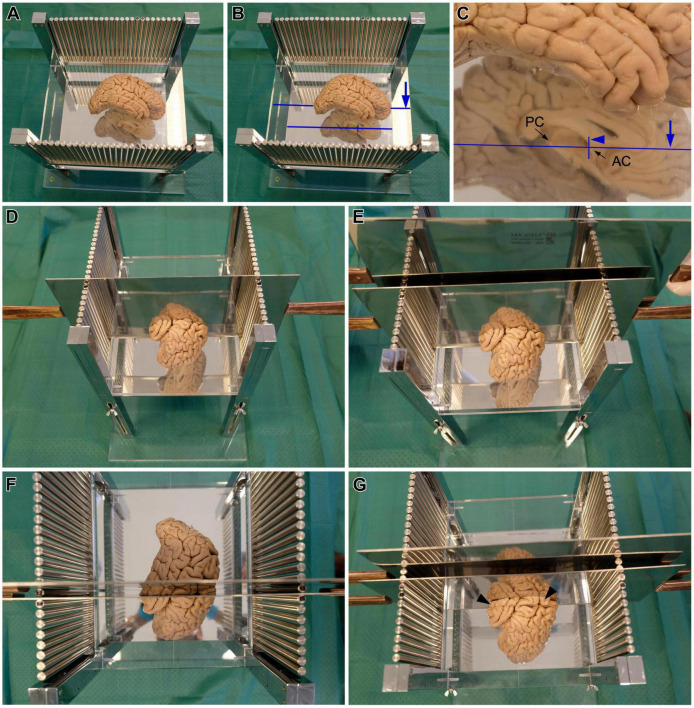
Steps for cutting *post-mortem* human brains in the stereotaxic instrument. **(A,B)** Brain hemisphere placed in the stereotaxic instrument, with the superior edge of the anterior commissure (AC) and the inferior edge of the posterior commissure (PC) aligned with the long line carved on the methacrylate plate [long blue line in panels **(B,C)**], and the posterior edge of the AC aligned with one the short lines carved on the methacrylate plate [short blue line in panels **(B,C)**]. **(C)** Detail of the AC and PC alignments. **(D)** A first blade is inserted in the slot between the metal columns corresponding with the position of the short carved line on the methacrylate plate, aligned with the posterior edge of the AC, which corresponds to coronal plane 0 of Talairach and Tournoux (1988). **(E)** A second blade is passed through the next slot toward the occipital pole, to obtain the coronal slab –1. **(F)** Superior view of coronal slab –1, in between the two blades. **(G)** The first blade is removed from the first slot and is introduced in the next slot toward the occipital pole, to obtain the coronal slab –2. Note the presence of coronal slab –1 in front of the blade (black arrowheads).

After fixation, either by immersion or perfusion, the *post-mortem* human brain is cut through the midline to separate the hemispheres. Brain hemispheres can be blocked together with the brainstem and cerebellum or the brainstem and cerebellum can be initially separated from the cerebrum by cutting through the interpeduncular fossa and the superior edge of the lamina quadrigemina. The two hemispheres are separated with a sagittal cut passing through the corpus callosum and the plane between the mammillary bodies, the frontal lobes, and the occipital lobes. This cutting is made from ventral to dorsal to have full view and control of the plane of cutting: one experimenter holds and stabilizes the brain with his/her hands on the dorsal surface of the hemispheres, exposing the ventral surface; then, another experimenter cuts between the hemispheres. Separating the two hemispheres is necessary because the AC and PC can only be identified in the sectioned midsagittal plane; AC and PC are the intracerebral references for stereotaxic blocking according to Talairach and Tournoux (1988). The medial face of one brain hemisphere is placed on the methacrylate plate facing the mirror (Figure 3A). Then, several hallmarks on the medial surface of the hemisphere are aligned with the carved lines on the methacrylate plate with the help of the mirror (Figures 2D, 3B, C). Specifically: the superior edge of the AC and the inferior edge of the PC are aligned with the long carved line on the methacrylate plate; also, the posterior edge of the AC is aligned with one of the two short carved lines (Figures 2D, 3C). Following these alignments, the hemisphere is oriented according to the stereotaxic space of Talairach and Tournoux (1988).

**FIGURE 4:**
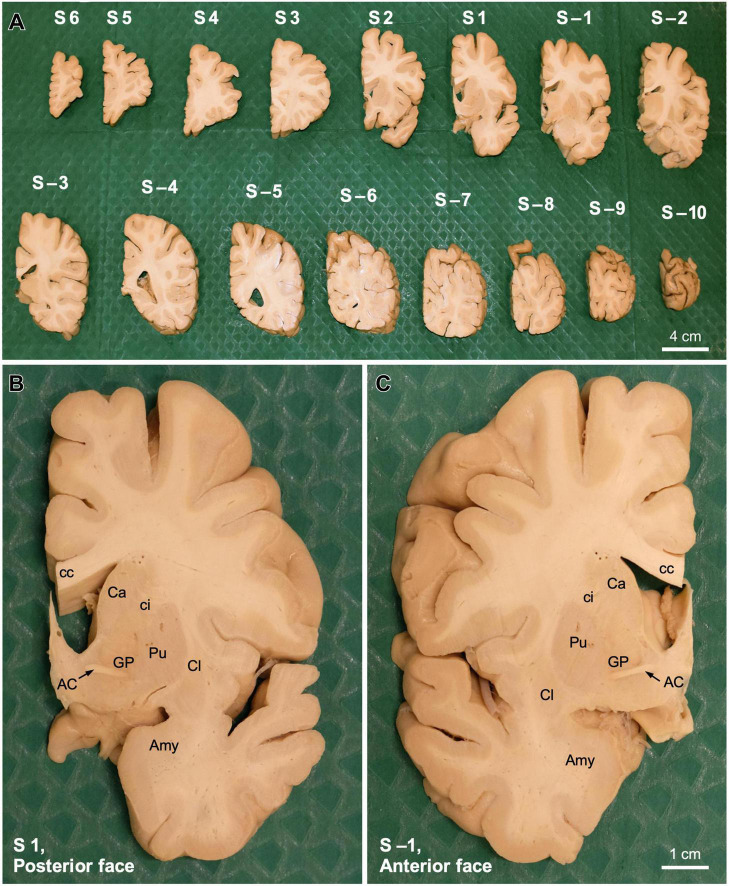
Coronal slabs from a human *post-mortem* brain obtained stereotaxically. **(A)** Coronal slabs obtained from the *post-mortem* human right hemisphere shown in Figure 3 using the stereotaxic instrument. Slabs are placed on the surgical drape on their anterior surface, thus their posterior surface is shown. Slabs are ordered from the frontal pole (S6, left upper corner) to the occipital pole (S –10, right lower corner). Slabs anterior/frontal to the coronal plane 0 are numbered with positive numbers (S1, S2… S6), while slabs posterior/occipital to the coronal plane 0 are numbered with negative numbers (S–1, S–2… S–10). **(B)** Posterior surface of coronal slab 1 (S1). **(C)** Anterior surface of coronal slab –1 (S –1). Note that the plane between panels **(B,C)** is the coronal anteroposterior plane 0. AC, anterior commissure; Amy, amygdala; Ca, caudate; cc, *corpus callosum*, ci, internal capsule; Cl, claustrum; GP, globus pallidus; Pu, putamen.

Once the hemisphere is aligned, a sharp blade is passed through the slot between the metal columns that corresponds with the short carved line aligned with the posterior edge of the AC (Figure 3D). This plane of cutting corresponds to coronal plane 0 of Talairach and Tournoux (1988). Then, another blade is passed through the next slot toward the occipital pole of the hemisphere (Figure 3E) to obtain a 1 cm thick coronal brain slab (Figure 3F); this slab is number −1 and its posterior surface is 1 cm from plane 0. The next step consists in removing the first blade and passing it through the next slot toward the occipital pole to obtain another 1 cm thick coronal brain slab (Figure 3G), whose anterior surface and posterior surfaces are 1 and 2 cm from plane 0, respectively. These procedures are repeated until the occipital pole is reached. The slabs cut posterior to the AC are numbered with negative values (Figure 4A).

After the posterior part of the hemisphere is cut, one blade is passed again through the slot corresponding to coronal plane 0. The same procedures described for cutting the part of the hemisphere posterior to the AC are repeated until the frontal pole is reached. Special care must be paid when cutting through the most anterior portion of the temporal pole that is not attached to the insula and the posterior orbitofrontal cortex because it does not hold with the reminder of the temporal lobe and can be squeezed by the blade. The slabs cut anterior to the AC are numbered with positive values (Figure 4A).

The instrument presented in this article is designed for stereotaxic *coronal* cutting of human brain hemispheres. However, cutting brains in the *horizontal* plane could be done with a similar instrument following analogous alignments: the long carved line on the methacrylate plate should run parallel to the planes of cutting through the slots, instead of perpendicular to them.

## 4. Results

The coronal slabs obtained from a *post-mortem* human brain hemisphere using the instrument and methods described in the preceding sections are shown in Figure 4A. Stereotaxic cutting as described in the present article yields coronal brain slabs oriented in the space of Talairach and Tournoux. Also, each and every slab, with the exception of those corresponding to the frontal and occipital poles and the loose part of the temporal pole, have the same thickness: 1 cm.

The stereotaxic slabs can be postfixed if fixation was not complete. Then, whole coronal slabs, or parts of them containing a region of interest (like the thalamus), that we name blocks, can be cryoprotected and cut on a freezing microtome to collect all the sections in consecutive series as described (García-Cabezas et al., 2007). Coronal plane 0 of the Talairach and Tournoux space is between the posterior surface of slab 1 (Figure 4B) and the anterior surface of slab −1 (Figure 4C). In these surfaces, AC is clearly visible (Figures 4B, C). Thus, the anterior surface of the first thin section obtained from microtome sectioning blocks from slab −1, starting on the anterior block face, at 50 μm thickness, corresponds to coronal plane 0 and its posterior surface corresponds to coronal plane −50 μm.

**FIGURE 5:**
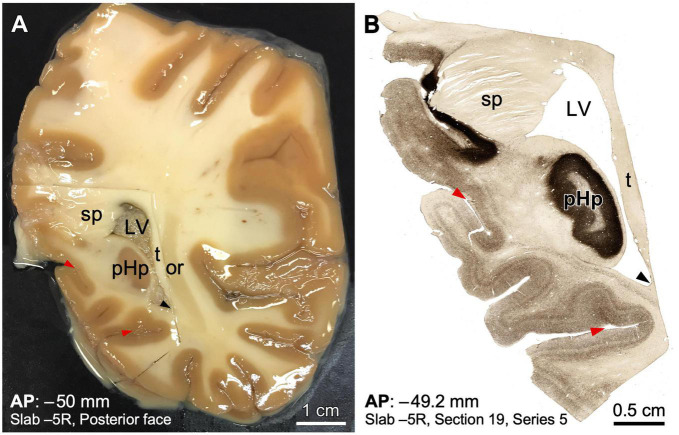
Example of human brain histological processing in known stereotaxic coordinates of the Talairach and Tournoux space. **(A)** Macroscopic picture of the posterior surface of slab –5 from a right human hemisphere. The region containing the posterior hippocampus has been dissected in a block for microtome sectioning and histological processing. The slab is soaked in an ethylene-glycol cryoprotective solution; thereby the glitter throughout (some prominent glitter has been toned down to improve visualization). **(B)** Microphotograph from a posterior view of a coronal section from the block shown in A processed for acetylcholinesterase, in an anteroposterior (AP) level near the posterior surface of the slab. Note that a precise stereotaxic AP coordinate is given, as well as the position of the section in the slab. Topographical references, such as sulci (red arrowheads) and ventricle angles (black arrowheads) have been indicated to facilitate comparison between the two photographs. LV, lateral ventricle; or, optic radiation; pHp, posterior hippocampus; sp, *splenium* of *corpus callosum*; t, *tapetum*.

We exemplify next the calculation of stereotaxic coordinates of a given histological section obtained from a stereotaxically cut *post-mortem* human brain. First, we select the brain slab with the region of interest and the block containing this region is separated from the rest of the slab (e.g., the posterior hippocampal formation at the level of the splenium of the corpus callosum; Figure 5A). Then, the block is cryoprotected and is freeze-sectioned in a freezing microtome, collecting the consecutive sections in the desired number of series. For example, to collect 10 series, each of the 10 first cut sections will be the first section in each of the ten series, which are collected separately. Then, section number 11 is section 2 of series 1 (and can be collected together with section 1 of the same series or on a separate well); section number 12 is section 2 of series 2, section number 13 is section 2 of series 3… and so on until section number 20, which is section 2 of series 10. Then, the procedure is repeated with section number 3 of each series (from cut section 21 to cut section 30). This serial sectioning is continued until the posterior face of the block is reached.

**FIGURE 6:**
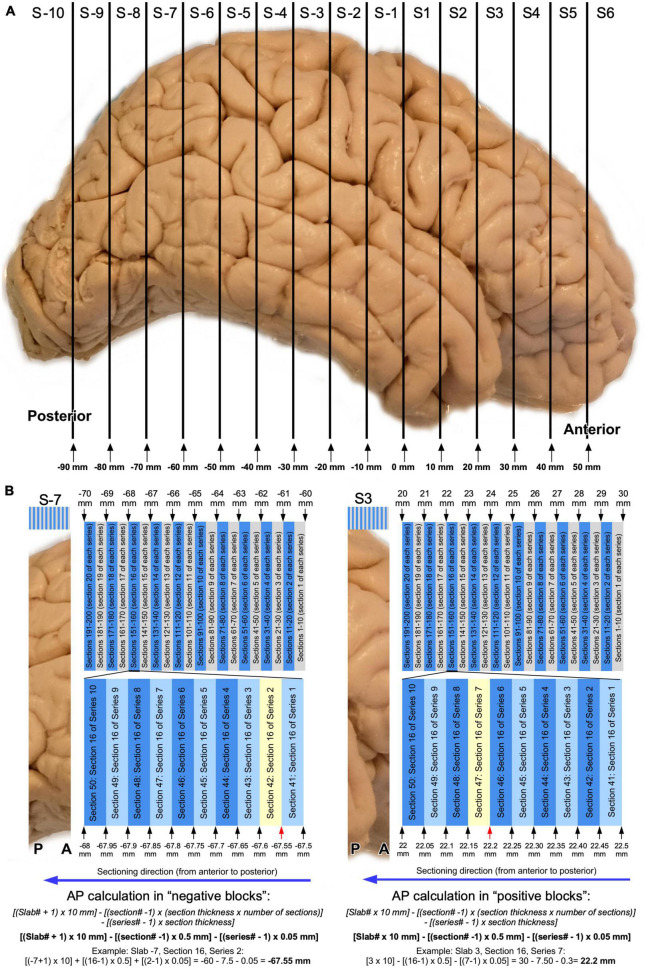
Graphic representation of AP level calculations of histological sections. **(A)** Schematic representation of slabs obtained from a right human hemisphere (Figures 3, 4). **(B)** Schematic representation of typical serial sectioning of a posterior slab (slab –7) with AP levels indicated in relevant positions: sections are 50 μm thick and 10 series are collected. Note than in this case, sections between the same series would be 500 μm (0.5 mm) apart. A random section as example is highlighted in yellow and AP calculations following the equation provided in the article is shown. **(C)** Typical serial sectioning of an anterior slab (slab 3) with AP levels indicated in relevant positions, and AP level calculated for a random section (highlighted in yellow).

Consequently, anteroposterior coordinates of any histological section from stereotaxically cut human brains can be calculated using several parameters: the section thickness, the series number, the section number, and the total number of series collected (Figures 5, 6). While cutting in the microtome, each section is labeled with a series and a section number. Because the section and series numbers are used later to calculate AP levels, we provide a formula to calculate these levels using section and series numbers, considering that the slabs are cut from anterior to posterior. The following equations provide the AP level of the anterior surface of any section (Figure 6):

For slabs anterior to AP 0: [Slab# × 10 mm]–[(section# −1) × (section thickness × number of series)]–[(series#–1) × section thickness].

For slabs posterior to AP 0: [(Slab# + 1) × 10 mm]–[(section# −1) × (section thickness × number of series)]–[(series#–1) × section thickness].

## 5. Discussion

Most articles on *post-mortem* human brain anatomy use thin brain sections obtained from manually cut brains (e.g., Hirai and Jones, 1989; Zilles et al., 1995). Actually, stereotaxic cutting of *post-mortem* human brains in the Talairach and Tournoux space (García-Cabezas et al., 2007; Pérez-Santos, 2021) is quite uncommon in contemporary human neuroanatomy. Nonetheless, earlier procedures for stereotaxic cutting of *post-mortem* human hemispheres have been described (Schaltenbrand and Bailey, 1959; Insausti et al., 1995). These procedures do not conform to the Talairach and Tournoux criteria, which is the most widely used in current neuroimaging. In line with this, the resourceful procedure described by Insausti et al. (1995) does not permit precise calculation of the stereotaxic coronal planes of actual brain slices, although it permits cutting human hemispheres consistently.

Precise stereotaxic cutting of *post-mortem* human brains is commendable because it provides slabs and blocks of human brain tissue than can be cut in histological thin sections of known position along the (anterior-posterior) axis perpendicular to the (coronal) plane of cutting. Thus, the coordinates in the space of Talairach and Tournoux on a given thin section can be known. This is useful, for example, to obtain maps of specific innervation (e.g., dopaminergic axons) in deep brain gray matter structures, like the thalamus (García-Cabezas et al., 2007). These maps can be easily collated with MR images obtained in the Talairach and Tournoux space, and permit extrapolation of *post-mortem* results to *in vivo* imaging.

The device described and illustrated in this article is proposed to neuroscientists working with *post-mortem* human brains, so that brain slices conform to the Talairach and Tournoux space, and data can be offered in this space. This approach should be particularly useful to compare experimental *post-mortem* data from human brains with *in vivo* neuroimaging data, as well as to make accurate comparisons of data from different laboratories/studies.

## Data availability statement

The original contributions presented in the study are included in the article, further inquiries can be directed to the corresponding author.

## Author contributions

CC developed the original conception and design of the stereotaxic device and read and corrected the manuscript. MG-C and IP-S wrote the first draft of the manuscript. All authors commented on previous versions of the manuscript, read, and approved the final manuscript.
